# Variation of inflammatory dynamics and mediators in primiparous cows after intramammary challenge with *Escherichia coli*

**DOI:** 10.1186/1297-9716-42-15

**Published:** 2011-01-24

**Authors:** Adel Pezeshki, Philippe Stordeur, Hugues Wallemacq, Frédéric Schynts, Mieke Stevens, Philippe Boutet, Luc J Peelman, Bart De Spiegeleer, Luc Duchateau, Fabrice Bureau, Christian Burvenich

**Affiliations:** 1 Department of Comparative Physiology and Biometrics, Laboratory of Genetics, Drug Quality and Registration Group, Ghent University, B-9000 Ghent, Belgium; 2 Departement Biotechnologie, plateforme Infectiologie Expérimentale, Centre d'Economie Rurale, B-6900 Marloie, Belgium; 3 Laboratory of Cellular and Molecular Physiology, GIGA-Research and Faculty of Veterinary Medicine, University of Liège, B-4000 Liège, Belgium

## Abstract

The objective of the current study was to investigate (i) the outcome of experimentally induced *Escherichia coli *mastitis in primiparous cows during early lactation in relation with production of eicosanoids and inflammatory indicators, and (ii) the validity of thermography to evaluate temperature changes on udder skin surface after experimentally induced *E. coli *mastitis. Nine primiparous Holstein Friesian cows were inoculated 24 ± 6 days (d) after parturition in both left quarters with *E. coli *P4 serotype O32:H37. Blood and milk samples were collected before and after challenge with *E. coli*. The infrared images were taken from the caudal view of the udder following challenge with *E. coli*. No relationship was detected between severity of mastitis and changes of thromboxane B2 (TXB2), leukotriene B4 (LTB4) and lipoxin A4 (LXA4). However, prostaglandin E2 (PGE2) was related to systemic disease severity during *E. coli *mastitis. Moreover, reduced somatic cell count (SCC), fewer circulating basophils, increased concentration of tumor necrosis factor-α (TNF-α) and higher milk sodium and lower milk potassium concentrations were related to systemic disease severity. The thermal camera was capable of detecting 2-3°C temperature changes on udder skin surface of cows inoculated with *E. coli*. Peak of udder skin temperature occurred after peak of rectal temperature and appearance of local signs of induced *E. coli *mastitis. Although infrared thermography was a successful method for detecting the changes in udder skin surface temperature following intramammary challenge with *E. coli*, it did not show to be a promising tool for early detection of mastitis.

## Introduction

The severity of coliform mastitis is of much more concern than its incidence [1]. Pathogen, cow and environment are three interdependent factors which influence the mastitis susceptibility [1]. From the various bacterial virulence factors studied during *Escherichia coli *mastitis [2], only a few have been found to play an important role in the outcome of the disease. It has been accepted that the type of *E. coli *strain is not the main factor in classification of severity. Preventive treatments which are efficient against contagious mastitis have been shown to be inefficient in the control of *E. coli *mastitis [3]. The severity of bovine *E. coli *mastitis is mainly determined by cow factors rather than by the pathogenecity of the invading pathogen and management [1]. It is known that the growth of *E. coli *in the udder cistern is specially related with the period of lactation and parity of cows. *E. coli *mastitis with severe clinical symptoms is more frequently observed around calving and during early lactation in dairy cows, whereas symptoms are mild to moderate during mid and late lactation. Because of hormonal, metabolic and nutritional alterations associated with pregnancy, immune system is compromised around calving (reviewed by Pezeshki et al. [4]). Cow parity is another important physiological factor that influences the severity of clinical coliform mastitis [5,6]. Clinical severe cases of coliform mastitis are mostly seen among multiparous cows rather than primiparous cows during early lactation. To our best knowledge the inflammatory status of primiparous cows ranking based on severity after intramammary infection of *E. coli *is poorly understood during early lactation. Physiological factors have been mainly studied in multiparous cows ranging from second lactation to sixth lactation [6-10].

Thromboxanes (TX), prostaglandins (PG), leukotriens (LT) and lipoxines (LX) which are the enzymatically generated products of cyclooxygenases (COX) and lipoxygenases are generated during inflammation and act as major pro-inflammatory mediators. There is evidence that TXB2 and PGE2 might have an important role in severity of *E. coli *mastitis in primiparous cows during early lactation [11]. Although not significantly, nonsteroidal anti-inflammatory drug treatment reduced the concentration of TXB2 and PGE2 in milk, while it had no effect on chemotactic inflammatory mediators and early innate immune molecules [11]. LTB4 is known to induce infiltration and sequestration of the neutrophils in the bovine mammary gland, whereas LXA4 serve as neutrophil "stop signals" in inflammation [12]. As excessive recruitment of neutrophils can exacerbate the inflammatory process via releasing detrimental intracellular products for tissues, persistence of LTB4 production or its ratio with LXA4 could be of particular importance in severity of the disease. We hypothesize that the magnitude of eicosanoids response plays an important role in the susceptibility to *E. coli *mastitis during early lactation in primiparous cows and can influence the resolution of this disease.

An automated method for early detection of coliform mastitis would prevent its progression and likely reduce the costs brought about by repeated treatments. Inflammation of the udder or mastitis results in increase of the temperature. The radiated heat emitted by the skin, reflecting subcutaneous circulation and tissue metabolism could be measured using infrared thermography. Although the thermography technique has been tested for early detection of experimentally induced acute clinical mastitis using *E. coli *endotoxin [13] or subclinical mastitis [14], the technique has not yet been validated for evaluating the temperature changes of the udder's skin surface after challenge with *E. coli*.

The objectives of the current study were to investigate (i) the outcome of experimental *E. coli *mastitis in primiparous cows during early lactation in relation with production of eicosanoids and inflammatory indicators in blood and milk and to determine (ii) whether thermography is a valid method for evaluating the temperature changes of the affected udder.

## Materials and methods

### Experimental animals and study facilities

In total, 9 primiparous Holstein Friesian cows in early lactation ranging in weight (one week after calving) from 511 to 615 kg were used. Cows arrived at the experimental dairy farm (Center of Rural Economy-CER-Marloie, Belgium) 15 days before expected calving date (actual day was 22 ± 6 days (d)). The age of cows was between 23 to 34 months at calving. Cows were milked twice a day at 06:30 and 18:30 using a quarter milking device [8]. After parturition, cows were fed a daily ration according to the National Research Council system (NRC, 2001). The forages were delivered in two equal meals at 07:00 and 19:00 while the concentrate was fed in several times, according to milk production. The composition and ingredient of the lactation diet used during the experiment is reported in Table 1. Water was provided *ad libitum*.

**Table 1:** Ingredient and nutrient compositions of the diet administered during the experimental period

Ingredient (Kg/d)	
Grass silage	18.50
Barley	1.00
Beet pulp	2.00
Commercial concentrate ^1^	8.00
Nutrient composition (%DM basis)	
DM (Kg/d)	17.18
NE_L_^2 ^(Mcal/kg)	1.55
CP	15.03
NDF	48.52
NFC^3^	25.66

^1 ^Contained 18% CP, 3% fat, 13% cellulose, 8000 UI/kg Vitamin A, 1200 UI/kg Vitamin D_3_, 15 mg/kg Vitamin E, 9.6 mg/kg CU_2_SO_4_.

^2 ^Calculated according to NRC (2001). NE_L _was estimated based on a cows weighing 650 kg and average 21.8 kg dry matter intake per day.

^3^NFC calculated as 100 - [(NDF - NDFCP) + CP + ether extract + ash] (NRC, 2001).

Experimental infections were approved by the Ethical Committee of the CER (No. META-DVA-1). After an adaptation period, animals were inoculated intramammarily with the *E. coli *suspension at 24 ± 6 days in milk. Bacteriological examination of the milk was carried out according to International Dairy Federation (IDF, 1981) recommendations. The cows were clinically healthy and free of major mastitis pathogens through three consecutive bacteriologically negative examinations, with a quarter foremilk somatic cell count (SCC) below 200 000 cells/mL. Daily quarter milk production (QMP) was recorded at d -7, -4, -3, -2, -1, 0, +1, +2, +3 and +6, relative to day of inoculation. On average, the concentration of preinfection β-hydroxybutyrate (BHBA), glucose and non-esterified fatty acids (NEFA) was 1.09 ± 0.42, 3.70 ± 0.42 and 0.33 ± 0.17 mmol/L, respectively.

### Bacteria and intramammary inoculation

The strain used in this study, to reproduce clinical signs of mastitis, was the *E. coli *strain P4, serotype O32:H37. The strain was maintained in a stock on lyophilization medium at -20°C. A stock of bacteria was sub-cultured in brain-heart infusion broth (CM225; Oxoid, Nepean, ON, Canada) during three consecutive days at 37°C. Subsequently, subcultures were washed 3 times with pyrogen-free PBS and resuspended in PBS [11]. Immediately before inoculation, a final concentration of 1 × 10^4 ^cfu/mL bacterial suspension in pyrogen-free PBS was prepared. On d0 (the day of challenge), 30 min after morning milking, the bacterial suspension was inoculated aseptically into the left quarter's teat cistern of all 9 cows by means of a sterile, pyrogen-free teat cannula (length, 7 cm, I.D., 2 mm; Me.Ve.Mat, Deinze, Belgium). The total volume of inoculation was 10 mL, consisting of 1 mL of bacterial suspension and 9 mL of pyrogen-free saline solution (NaCl 0.9%; Baxter N.V., Lessines, Belgium) per quarter [5,15].

### Sampling procedure

Blood samples were collected in the morning on d -2, +1, +2, +3, +6 relative to the day of challenge. On the day of challenge, blood samples were collected at -0.5, +0.5, +3, +6, +9, +12, +15, +18, +21 hours (h) relative to the time of challenge. Blood samples (3 × 9 mL) were drawn aseptically from the external jugular vein by venipuncture into 3 vacutainer tubes (BD Biosciences, USA) containing Lithium-Heparin (10 IU/L), EDTA or no anticoagulant. Plasma was separated from the blood samples in tubes containing Lithium-Heparin and stored at -20°C until analysis of metabolites. Serum was harvested from blood samples in dry tubes and stored at -20°C for later analysis of haptoglobin. The blood samples in tubes containing EDTA were used for the determination of hematologic parameters.

Quarter milk samples were taken on d -7, -4, -2, +1, +2, +3, +6 relative to the day of challenge. On the day of challenge, milk samples were collected at -0.5, +0.5, +3, +6, +9, +12, +15, +18, +21 h relative to the time of challenge. Equal volumes of milk samples were collected for each specific analysis within first 12 h after the challenge and at other time points mentioned above. Foremilk (5 mL) was aseptically collected for quantification of *E. coli *(cfu/mL), SCC and milk composition and stored at 4°C until analysis. Morning milk samples (20 mL) were collected for determination of eicosanoids and stored at -70°C prior to analysis. The collected morning milk samples (5 mL) for quantification of ions were stored at -20°C before the analysis begins.

### Clinical examinations

The cows were examined and scored generally for rectal temperature, skin turgor, rumen motility, general attitude [5] and specifically for udder temperature, quarter swelling, pain, teat relaxation, milk leakage and milk appearance [11] until six days after the intramammary inoculation of *E. coli*.

### Infrared thermographic imaging

The infrared images were taken from the caudal view of the experimental and control quarters at 0, 3, 6, 9, 10, 11, 12, 13, 14, 15 PIH. To ensure integrity of the thermal data and reduce the effect of environmental factors, some considerations were standardized in the present study: (i) all infrared images were captured within the stall and (ii) at the same distance (1.5-2 m) from the subject, when (iii) cows were in rest with no exercise. (iv) Circadian rhythm effects were controlled by scanning the animals at the same time of the day. Moreover, any extraneous debris and foreign materials like manure, straw and moisture were removed from the body surface to have clear infrared images from the skin of the udder. A hand-held portable infrared camera (ThermaCAM^® ^E2, FLIR SYSTEMS) with thermal sensitivity of 0.12°C was used to take images of all animals. The camera was calibrated to ambient temperature and absorptive conditions on each scanning time. An emissivity value of 0.93 was set on the camera before performing the scanning.

### Analytical methods

The concentration of milk eicosanoids was determined with a commercially available competitive ELISA kit (Neogen, Lexington, KY, USA). Briefly, quarter milk samples were filtered using a 70-μm cell strainer (Becton Dickinson; Erembodegem, Belgium) to discard cell clusters. Milk sample was diluted with distilled water (50:50 v/v), and 1 mL of the mixture was loaded on a conditioned 100-mg C_18 _column (Varian, St.-Katelijne-Waver, Belgium). After washing the column subsequently with distilled water, methanol: distilled water (30:70 v/v), and hexane, the column was centrifuged at 3200 × *g *for 3 min to remove any trace of hexane. Finally, eicosanoids were eluted from the C_18 _column with 1 mL of methanol. The collected elute was dried under a stream of nitrogen. Dried samples were reconstituted in an appropriate volume of assay buffer and manufacturer's instructions were followed for measuring the concentration of eicosanoids.

The number of *E. coli *(cfu/mL) in milk was determined by plating out the prepared dilutions in PBS on drigalski agar (Biokar Diagnostic, Beauvois, France) and counting the colonies after 24 h of incubation at 37°C. Milk sodium, chlorine and potassium were measured using an ion-selective electrode analyzer (Ilyte; Instrumentation Laboratories, Milan, Italy). Milk SCC was determined using a fluoropto electronic method (Fossomatic 400 cell counter; Foss Electrics, HillerØ, Denmark). Milk fat, protein and lactose were determined using mid-infrared-photospectrometry (MilkoScan 4000; Foss Electrics, HillerØ, Denmark). Total blood leukocyte count was determined using an electronic particle counter (Coulter Counter Z2; Coulter Electronics Ltd., Luton, UK). Differential blood leukocyte count was performed on blood smears using bright field microscopy as previously described [16]. Plasma tumor necrosis factor-α (TNF-α) and Interleukin-1β (IL-1β) were quantified with ELISA assays as previously described [17]. Mouse anti-recombinant bovine TNF-α and mouse anti-ovine IL-1β antibody were commercially available (Serotec Ltd., Oxford, UK). Serum was assayed for haptoglobin using the hemoglobin-binding assay method. Blood metabolites were analyzed at 37°C using a clinical auto-analyzer (ILAB 600, Instrumentation Laboratory, Lexington, MA, USA).

### Infrared images

**Figure 1:**
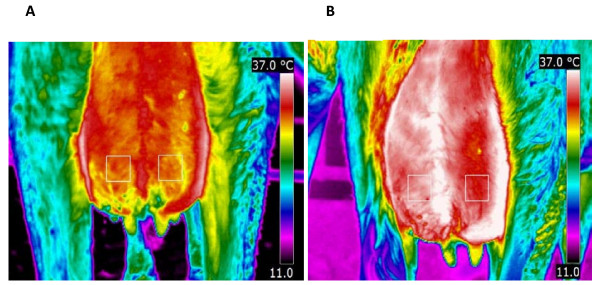
**Thermal images of the caudal part of the udder of a primiparous cow after challenge with *E. coli *strain P4, serotype O32:H37 (1 × 10^4 ^cfu) at post infusion hour (PIH) 3 (A) and PIH 12 (B) during early lactation**. Two fixed 25 × 25 pixel areas above the teats used to extract the data at various time points following intramammary infection. Left quarters were infected at PIH 0 and right quarters were control quarters. There was a significant increase for udder skin temperature between PIH 0 and PIH 12 in both infected (*P *= 0.02) and control quarters (*P *= 0.009).

The output of the infrared camera was un-calibrated digitized images with pixel points. Pixel points were analyzed with appropriate computer software (FLIR QuickReport 1.2) to generate mean temperature of the skin surface. The mean temperature variation of four different areas between the groove of the udder and the hind leg and median suspensory ligament on caudal udder skin surface was examined. The areas were selected above the infected and control teats and centered according to the teats. The mean temperature of rectangles of 25 × 25 pixels drawn by the help of the camera software was measured (Figure 1). The two areas just above the teats showed the smallest variation in the temperature of the udder (Figure 1). An example of the infrared images at 3 and 12 PIH is shown in Figure 1A and Figure 1B, respectively.

### Statistical analysis

The main objective was to study the disease severity in relation with production of different inflammatory mediators/indicators and clinical parameters. Disease severity was defined as the percentage decrease of QMP in uninfected quarters at 48 PIH compared to QMP in the same quarters at -24 PIH [8]. To investigate the relationship of various parameters analyzed in milk and blood and disease severity, a mixed model was used with cow as random effect, and time, disease severity and their interaction as fixed effects. To explore the relationship between disease severity and systemic and local inflammatory responses, the Kendall's correlation coefficient between categorical parameters and disease severity was calculated. To study the kinetics for temperature of udder skin in infected and uninfected quarters, a mixed model was used, with temperature as response variable, cow as random effect, and PIH, udder point and their interaction as categorical fixed effects. To compare the measurements at different time points with time zero, the mixed model was used, with measured parameters as response variable, cow as random effect and PIH as categorical fixed effect. For milk production, measurements at all times were compared with time -48. Significance level for multiple comparisons was adjusted by Dunnett's procedure. A *P *value of < 0.05 was considered significant. Values in the graphs shown are mean ± SEM.

## Results

### Preinfection milk and blood constituents and disease severity

A relationship was found between preinfection SCC and disease severity (*P *= 0.002), with higher preinfection SCC corresponding to higher disease severity. We failed to detect a significant relationship between preinfection milk fat, protein, lactose, and milk production and disease severity. No correlation was found between the severity of experimental coliform mastitis and preinfection concentrations of BHBA, glucose and NEFA.

### Disease severity and local and systemic inflammatory response

**Figure 2:**
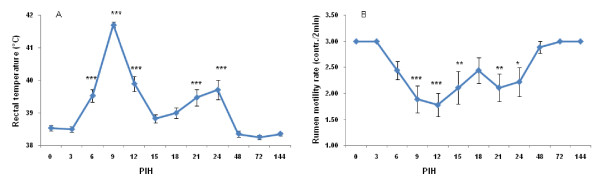
**Rectal temperature (A) and rumen motility rate (B) of 9 primiparous cows challenged intramammarily in 18 quarters (left quarters) with 1 × 10^4 ^cfu *E. coli *strain P4, serotype O32:H37 at post infusion hour (PIH) 0 during early lactation**. Means (± SEM) are shown. The clinical symptoms were scored according to Vangroenweghe et al. [5].* *P *< 0.05, ** *P *< 0.01, ***P < 0.001: significance of difference from the control value.

Following intramammary *E. coli *inoculation, rectal temperature (RT), skin turgor score and general attitude score rapidly increased to reach a maximum at PIH 9. There was a relapse of RT and RT increased up to 39.7°C again at PIH 24 (Figure 2A). There was no relationship between disease severity and RT changes. Rumen motility was reduced in all cows from PIH 3 onward (Figure 2B). The depression of reticulorumen motility was more pronounced at PIH 9, with 7 animals showing a slight decrease in motility (1 to 2×/2 min); one animal showed a complete absence of reticulorumen activity. Reticulorumen motility was normal for all cows PIH 48 onward. A significant negative correlation was found between the rumen motility rate and disease severity (r = -0.56; *P *= 0.04).

Local clinical signs at the level of the infected mammary gland were also investigated. Cows became restless and showed symptoms of pain. The first changes in milk appearance (flakes, color, etc.) occurred at PIH 9, with two animals showing some flakes in milk. Two most severe responders showed more flakes and clots and slightly watery milk at PIH 48 and 72. Although milk appearance was normal for all moderate responders at PIH 144, watery milk with more flakes and clots was observed for the most severely diseased cow at this time point. Slight swelling of the infected quarters occurred in 3 cows at PIH 3. Seven cows showed moderate swelling of the infected quarters at PIH 9. Pronounced swelling disappeared gradually, and was already minimal (8 quarters with slight swelling) at PIH 48. Teat relaxation and milk leakage were only observed in a small number of cows.

### Infrared thermography

**Figure 3:**
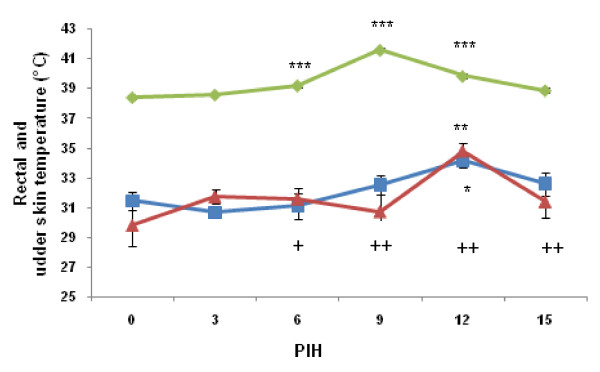
**Rectal temperature (lozenge) and udder skin temperature of the infected (square) and control quarter (triangle)**. Swelling of infected quarters: +mild, ++moderate. See legend of Figure 2 for more details.

The temperature of the udder skin on the infected quarter was significantly higher than that of the control quarter (*P *= 0.04). The mean udder temperature of infected quarters was increased from 3 PIH onward (Figure 3). However, skin temperature of the control quarters was increased from 9 PIH onward only. A peak of udder skin temperature in infected and control quarters occurred after the peak of rectal temperature (12 vs. 9 PIH, respectively). The temperature of udder skin at 12 PIH for both infected and uninfected quarters was significantly higher compared to 0 PIH (*P *= 0.02 and *P *= 0.009, respectively). There were no significant differences between udder skin temperatures at other time points when compared to 0 PIH. Thereafter, the udder skin temperature returned to normal in both quarters.

### Disease severity and milk eicosanoids

**Figure 4:**
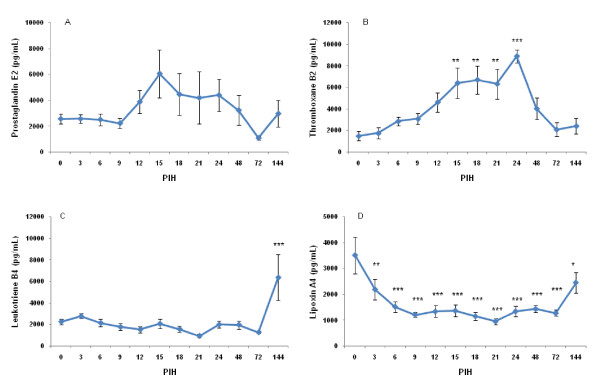
**Prostaglandin E2 (A), thromboxane B2 (B), leukotriene B4 (C) and lipoxin A4 (D) in secreta of all infused quarters at various times following intramammary infection**. See legend of Figure 2 for more details.

In milk, the mean PGE2 concentration immediately after intramammary inoculation was 2572.9 ± 1155.6 pg/mL. Overall, there was a significant time effect for PGE2 (*P *< 0.01; Figure 4A). There was a relationship between disease severity and PGE2 concentration (*P *< 0.0001), with higher PGE2 concentrations corresponding to higher disease severity. The mean concentration of milk TXB2 immediately after infusion of *E. coli *was 1504.3 ± 1267.7 pg/mL. After infusion of *E. coli*, TXB2 changed significantly over time (*P *< 0.0001; Figure 4B). TXB2 started to increase from PIH 9 onward and returned to its normal level after PIH 24. Immediately after infusion of *E. coli*, the mean concentration of LTB4 was 2270.7 ± 776.5 pg/mL. There was no significant effect of time for concentration of LTB4 in milk (Figure 4C). In milk, the mean concentration of LXA4 immediately after infusion was 3513.9 ± 2108.1 pg/mL. The effect of time on the concentration of LXA4 was significant (*P *< 0.0001; Figure 4D). LXA4 decreased gradually after intramammary infusion of *E. coli *and did not normalize before 144 PIH. We failed to detect any relationship between disease severity and concentrations of TXB2, LTB4 and LXA4.

### Disease severity and indicators of inflammation in milk and blood

**Figure 5:**
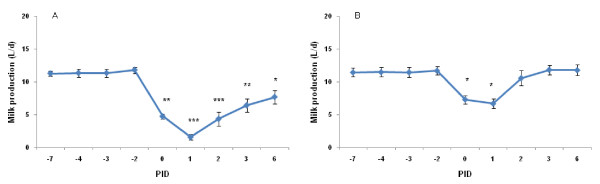
**Milk production of the infected (A) and uninfected quarters (B)**. See legend of Figure 2 for more details. PID: post infusion day.

The infected and uninfected QMP of cows used in the current study is shown in Figure 5A and 5B, respectively. In the infected quarters, QMP decreased on d 0, the day of intramammary inoculation (Figure 5A). Infected QMP decreased the lowest level on d+1, relative to day of challenge. On d+3, the recovery in the infected quarters was not complete compared to the preinfection.

The effect of time on the number of *E. coli *in inoculated quarters was significant (*P *< 0.0001). Overall, the number of *E. coli *increased to peak values of 4.96 log_10 _cfu/100 mL at PIH 6. No significant correlation was found between disease severity and the number of *E. coli*. SCC increased following the intramammary *E. coli *infection. There was a significant relationship between disease severity and SCC, with lower SCC values observed for more heavily diseased cows (*P *= 0.03).

Following the intramammary challenge, marked changes in number of blood neutrophils (*P *< 0.0001), basophils (*P *< 0.0001) and eosinophils (*P *= 0.005) happened over time. The number of neutrophils started to decrease from PIH 6 onward, with nadir at PIH 9. The number of circulating neutrophils increased after PIH 9 and reached initial values by PIH 24. The number of circulating eosinophils decreased gradually after PIH 3 to reach its minimal level at PIH 24. There was a significant relationship between disease severity and the number of basophils (*P *= 0.003), with more diseased cows having lower basophil levels. Following intramammary challenge, marked changes in the number of lymphocytes and monocytes were observed (*P *< 0.0001). Although not significantly, lymphocytes showed a temporary increase at PIH 3 and decreased from PIH 6 onward, with nadir at PIH 12. The number of lymphocytes gradually recovered from PIH 24 onward. Immediately after intramammary inoculation of *E. coli*, the number of monocytes started to decrease and reached its lowest level at PIH 9. The number of monocytes increased from PIH 9 onward until the end of the study. There were significant differences in the numbers of white blood cells (WBC) over time (*P *< 0.0001). WBC decreased from 6 PIH onward, reached its nadir at PIH 12 and started to return to its normal level from PIH 24 onward. Kinetic of the WBC was not related significantly with disease severity.

**Figure 6:**
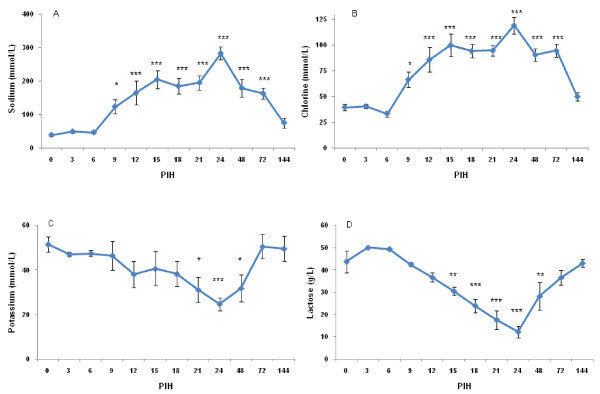
**Sodium (A), chlorine (B), potassium (C) and lactose (D) in secreta of all infused quarters at various times following intramammary infection**. See legend of Figure 2 for more details.

After intramammary inoculation of *E. coli*, marked changes in concentration of indicators for the disintegration of the blood-milk barrier (i.e. milk sodium, chloride, potassium and lactose) occurred (Figure 6A, B, C, D). Following the *E. coli *challenge, the lactose concentration started to decrease after PIH 6, reaching nadir at PIH 24. Changes of chlorine and lactose were not related to disease severity. However, there was a significant relationship between disease severity and potassium (*P *< 0.0001) and sodium (*P *= 0.03) levels, with more diseased cows having higher sodium and lower potassium in milk. Milk fat and protein was changed over time following intramammary inoculation of *E. coli *(*P *< 0.0001).

The kinetics of plasma TNF-α and IL-1 did not differ significantly over time after the challenge. Following infusion of *E. coli*, a significant relationship between disease severity and concentrations of TNF-α (*P *< 0.0001) and IL-1 (*P *< 0.0001) was found. More severely diseased cows had lower IL-1 but higher TNF-α levels. The concentration of haptoglobin changed significantly over time (*P *< 0.0001). Serum haptoglobin in the infected quarters increased from PIH 15 onward, peaked at PIH 72 and returned to its initial levels at PIH 144. No significant relationship between disease severity and the concentration of haptoglobin was detected.

## Discussion

As expected, similar to animals receiving the same dose (1 × 10^4 ^cfu) of *E. coli *in previous experiments [5,15], quarter swelling, fever, depression of reticulorumen motility, changed milk appearance and presence of pain in udder was observed following intramammary *E. coli *challenge. In line with results of Vangroenweghe et al. [5,11], the variation of clinical responses in primiparous cows was quite small in this study. The preinfection concentration of BHBA, NEFA and glucose indicated that the cows in the present study were not ketotic at the time of challenge [9]. In contrast to previous research [9,10], no correlation existed between the severity of experimental *E. coli *mastitis and preinfection concentrations of BHBA, glucose and NEFA. The clinical outcome of the disease in terms of severity was determined using a day-to-day method [8,10] (i.e. QMP loss in uninfected quarters) in the current study.

We quantified TXB2, PGE2, LXA4 and LTB4 in the milk of cows challenged intramammarily with *E. coli *and studied the relationship between these variables and severity of mastitis. The eicosanoids are derived from arachidonic acid and are important mediators and modulators of inflammation. They are released locally during coliform and LPS mastitis [18-20]. We showed that milk PGE2 and TXB2 concentrations were increased after intramammary challenge of *E. coli*. In the present study, the concentration of PGE2 was linked to the severity of mastitis; however there was no relationship between severity of the disease and changes of TXB2, LXA4 and LTB4. A significant increase in PGE2 [20] and TXB2 [18-21] has been reported in milk after intramammary *E. coli *challenge. Although Vangroenweghe et al. [11] could not detect any significant difference in milk PGE2 and TXB2 concentrations between carprofen- and the saline-treated animals, the concentration of TXB2 and PGE2 remained lower in carprofen-treated animals with more moderate responses. The severity of *E. coli *mastitis seems to be linked to the activity of specific key enzymes, i.e. COX that are involved in the synthesis of prostaglandins and thromboxanes. Schmitz et al. [22] demonstrated that the mRNA synthesis of COX-2 was increased in LPS-treated quarters and reached its highest level 3 h after infusion of LPS. The activity of this enzyme in connection to severity of coliform mastitis remains to be investigated in primiparous cows during early lactation.

In the present study, an attempt was made to gain further insights into the possible interrelationships between the various indicators of inflammation and severity of experimentally induced *E. coli *mastitis in primiparous cows during early lactation. The negative correlation found between rumen motility rate and severity of the disease could be due to elevated concentrations of TNF-α in the blood plasma of severely diseased cows. A positive relationship was found between depressed circulating basophils and severity of coliform mastitis. No significant association between the number of total white blood cells, neutrophils, eosinophils, lymphocytes and monocytes and severity might be explained by the limited number of animals in this study. The recruitment of neutrophils into the challenged quarters as reflected by SCC is an important aspect of the defense mechanism against *E. coli*/endotoxin mastitis. We found a negative relationship between SCC and severity of the disease. It is presumed that the lower SCC is associated with a relative slow influx of PMN and clearance of the bacteria from the glands of the most severe cases of coliform mastitis. In contrast to previous research [10], the severity of coliform mastitis was not linked to the number of bacteria that grow in the gland cistern in the present study.

Our results showed that the thermal camera was successful in detecting the 2-3°C changes in temperature of the udder skin associated with clinical mastitis. Similarly, previous research demonstrated that a thermal camera is capable of detecting 1-1.5°C [13] and 2.3°C [23] temperature changes of udder surface of cows intramammarily infused with endotoxin. In contrast with their results, the temperature of the udder skin of the experimental and control quarters was not increased simultaneously with rectal temperature and there was a delay in the peak of udder skin temperature in our study. The coincidence of increase in skin temperature with decrease in body temperature is probably due to peripheral cutaneous vasodilation during defervescence (abatement of fever). In fact, the generated heat during inflammation is transmitted to the skin surface through augmented capillary blood flow and dissipated as infrared energy [24]. As a result of increased permeability of capillaries, plasma leaked into the interstitium and maximal swelling occurred between PIH 9 and 15 (Figure 1B). In an earlier study [25], we demonstrated that after infusion of LPS, the increase in blood flow showed a biphasic aspect, both in goats and cows, with a return to normal values between the 2 peaks. It appears that the peak observed for udder skin temperature in the current study coincides with the second peak in the study of Dhondt et al. [25]. We found that the temperature of udder skin is increased both in experimental and control quarters which was in accordance with results of Hovinen et al. [13]. Scott et al. [23] demonstrated a clear rise in temperature of the infected quarters and a lower rise in the control quarter 1 h after inoculation of LPS. Increase of udder temperature in the infected and control quarters reflects a systemic effect of *E. coli*. The thermal camera showed increased udder skin surface temperature at PIH 12 which was later than the appearance of systemic and local signs at PIH 9. Therefore, infrared thermography was not a successful method in early detection of experimentally induced *E. coli *mastitis.

In conclusion, intramammary PGE2 production was related to bacterial growth and intensity of the inflammatory reaction. This is in agreement with the general concepts of acute inflammation. Moreover, severity of *E. coli *mastitis in primiparous cows was linked to SCC, circulating basophils, concentration of plasma TNF-α and milk sodium and potassium concentrations which might be the result of underlying mechanisms which are responsible for individual variation in the susceptibility to *E. coli *mastitis. Infrared thermography was a successful technique to detect the changes in temperature of the udder skin surface in both infected and control quarters after challenge with *E. coli*. However, this increase in udder skin surface temperature recorded by thermal images occurred after the appearance of local signs, indicating that the infrared thermography technique was not promising for early detection of *E. coli *mastitis. The increase in skin temperature coincided with the decrease in body temperature which is probably the result of the peripheral cutaneous vasodilation during defervescence.

## Competing interests

The authors declare that they have no competing interests.

## Authors' contributions

AP carried out the field study and drafted the manuscript. PS carried out the field study. HW participated in the sample analysis. FS provided the facilities in the experimental farm. MS participated in scientific discussions of the results. PB participated in the sample analysis and scientific discussions. LJP and BDS participated in design of the study and coordination. LD performed the statistical analysis. FB provided the facilities for sample analysis. CB designed the study and participated in the scientific discussions. All authors read and approved the final draft.
